# Self-assembled IR780-loaded transferrin nanoparticles as an imaging, targeting and PDT/PTT agent for cancer therapy

**DOI:** 10.1038/srep27421

**Published:** 2016-06-06

**Authors:** Kaikai Wang, Yifan Zhang, Juan Wang, Ahu Yuan, Minjie Sun, Jinhui Wu, Yiqiao Hu

**Affiliations:** 1 State Key Laboratory of Pharmaceutical Biotechnology, Medical School of Nanjing University, Nanjing 210093, China; 2 State Key Laboratory of Natural Medicines, China Pharmaceutical University, Nanjing 210009, China; 3 Institute of Drug R&D, Medical School of Nanjing University, Nanjing 210093, China; 4 Jiangsu R& D Platform for Controlled & Targeted Drug Delivery, Nanjing University, Nanjing 210093, China

## Abstract

Combination of photothermal and photodynamic therapy (PTT/PDT) offer unique advantages over PDT alone. However, to achieve synergetic PDT/PTT effect, one generally needs two lasers with different wavelengths. Near-infrared dye IR-780 could be used as photosensitizer both for PTT and PDT, but its lipophilicity limits its practical use and *in vivo* efficiency. Herein, a simple multifunctional IR780-loaded nanoplatform based on transferrin was developed for targeted imaging and phototherapy of cancer compatible with a single-NIR-laser irradiation. The self-assembled transferrin-IR780 nanoparticles (Tf-IR780 NPs) exhibited narrow size distribution, good photo-stability, and encouraging photothermal performance with enhanced generation of ROS under laser irradiation. Following intravenous injection, Tf-IR780 NPs had a high tumor-to-background ratio in CT26 tumor-bearing mice. Treatment with Tf-IR780 NPs resulted in significant tumor suppression. Overall, the Tf-IR780 NPs show notable targeting and theranostic potential in cancer therapy.

Photodynamic therapy (PDT) is a promising tumor-ablative medical intervention under development for a broad range of applications in the field of oncology^1^,^2^,^3^. The PDT depends on the laser-induced capability of photosensitizers (PSs) to transfer energy to oxygen dissolved in tumor environment to produce cytotoxic singlet oxygen (^1^O_2_), that subsequently causes induction of cell death and necrosis of proximal tissues^4^. However, the PDT efficacy of solid tumors is largely limited by issues including: (1) hydrophobic nature of most PSs, which leads to easy aggregation in aqueous media and negative effects on their photophysical, chemical and biological properties^5^,^6^; (2) the excitation wavelength below 700 nm, which limits permeation distance and causes insufficient photocytotoxicity^7^,^8^,^9^,^10^; (3) low production of singlet oxygen due to severe hypoxia caused by the oxygen consumption and vascular shutdown in tumors^11^,^12^.

To overcome the current limitations of PDT, new synergistic treatment modalities have been adopted which combine PDT with other therapies such as photothermal therapy (PTT). For example, nanoparticles (NPs) such as carbon nanohorns^13^, gold vesicles (GVs)^14^, and graphene oxide^15^ complexed with PSs on their surfaces are used to enhance the tumor accumulation of PSs and production of heat and singlet oxygen for synergistic PDT/PTT. However, PDT/PTT based on photothermal coupling agents generally needs two lasers with different wavelengths due to the absorption mismatch of PSs and photothermal agents. The sequential irradiation prolongs the treatment time and requires precise alignment of the two light beams. Therefore, there still remains great challenge to develop a simple and effective therapeutic strategy for simultaneous synergistic PDT/PTT treatment.

NIR dyes are being used as promising imaging and therapeutic agents for the treatment of tumors by PTT or PDT^16^. Indocyanine green (ICG) is a tricarbocyanine NIR dye which has absorption and emission maxima around 780 and 830 nm in the NIR region with low absorptivity by tissue chromophores^17^. However, ICG therapeutic use is limited by various factors, such as poor *in-vitro* aqueous stability, concentration-dependent aggregation behavior, short circulation half-life, and off-target effects^18^. IR-780 iodide is a more recently developed NIR dye which is more stable than ICG. IR-780 is a lipophilic cationic heptamethine dye with higher fluorescence intensity than ICG^19^,^20^,^21^. Currently, IR780 iodide has been reported to have the ability of producing singlet oxygen under irradiating at wavelength of 808 nm, which can be used for PDT^22^. Meanwhile, IR780 can be also used as a PTT agent because of generation of heat upon laser irradiation^20^. Despite the promise, the clinical use of IR780 iodide is limited because of its poor aqueous solubility and low tumor-targeting efficacy.

Here, we developed a simple, but effective, therapeutic strategy for simultaneous synergistic PDT/PTT treatment, using transferrin as a drug carrier to load IR780 iodide, forming Tf-IR780 NPs which can be excited with 808 nm laser to generate heat and singlet oxygen, thus destroying cancerous cells *in-vitro* and *in-vivo* via its photosensitizing property. In many ways, transferrin represents an ideal carrier for drug delivery^23^,^24^. It binds to the transferrin-receptor (TfR) with high affinity and transports iron into cells^25^. Due to increased need for iron in tumor cells, the overexpression of TfR has been confirmed in multiple human tumors, including breast and prostate cancer^26^. Tf-mediated drug delivery has been extensively used for tumor targeting for several decades^27^,^28^,^29^. Our work extends the previous work by using Tf not only for its targeting ability but also as a drug delivery carrier. The developed system represents a simple, safe, and imageable nanoprobe which can be used for combination PDT/PTT treatment.

## Materials and Methods

### Materials

Human transferrin, IR780 and 2′,7′-dichlorodihydrofluorescein diacetate (H2DCFDA) were obtained from Sigma-Aldrich (USA). Dithiothreitol (DTT), dimethyl sulfoxide (DMSO), and 4% paraformaldehyde were purchased from NanJing WanQing Chemical Glassware Instrument Co. Ltd (China). The cell counting kit-8 (CCK-8) was supplied by Dojindo Laboratories (Japan). Singlet oxygen sensor green (SOSG), Calcein-AM and Propidium Iodide (PI) Cell Apoptosis Kit were obtained from Invitrogen (USA). Deionized water was used throughout the experiments.

### Preparation of Tf-IR780 NPs

Tf-IR780 NPs were prepared by a modified “molecular switch” method reported previously. Briefly, Tf was dissolved in water (2 mg/mL) and mixed with DTT for 5 minutes. Then, IR780 solution (3 mg/mL in ethanol) was added with gentle stirring. The formed Tf-IR780 NPs were extensively dialyzed with a membrane with MW cut-off 8 kDa to remove any remaining DTT and free IR780. The product was isolated by freeze-drying and stored in a freezer for further use. The content of IR780 in Tf-IR780 NPs was determined by UV–vis absorption spectroscopy using IR780 standard curve.

### Characterization of Tf-IR780 NPs

The particle size and zeta potential were measured at room temperature by dynamic light scattering (DLS, 90Plus, Brookhaven Instrum. Corp). The morphologic and particle size examination of the Tf-IR780 NPs was further conducted by transmission electron microscopy (TEM, JEM-2100, Japan).

The absorptions of free IR780 and Tf-IR780 NPs dispersed in water were measured on an UV/vis spectrophotometer (UV-2450, Shimadzu, Japan) with a quartz cell with a 1 cm path length.

The release study of Tf-IR780 NPs at different pH values was performed. Predetermined amount of lyophilized nanoparticles were dissolved in 1 mL of phosphate buffer solution (PBS, 0.01 M, PH 7.4) and put into a dialysis bag (MWCO 3500). Then the bag was immerged into 15 mL release medium (PBS containing 0.1% v/v Tween 80) at different pH values (pH5.5, pH6.8 and pH7.4), and kept in an incubator at 37 °C. At predetermined time intervals, the release medium was withdrawn for UV–vis analysis. The amount of the IR780 was determined by UV–vis absorption spectra according to standard curve.

### Photostability of Tf-IR780 NPs

Tf-IR780 NPs (2 mg in 3 mL water) were irradiated with 808 nm laser at 1 W/cm^2^ for 0, 30, 60, 120, 180, and 300 s. The absorption spectra of Tf-IR780 NPs and free IR780 dissolved in DMSO were measured immediately after dilution with water and after 1-day storage under ambient light using the UV–vis spectrophotometer.

### Photothermal performance in solution

Tf-IR780 NPs were diluted to 0.05 mg/mL and exposed to an 808 nm NIR laser at a power density of 1 W/cm^2^ for 5 min. A thermometer was used to measure the temperature every 30 s. Free IR780 and PBS were used as controls.

### Singlet oxygen generation

Singlet oxygen sensor green (SOSG) was employed to evaluate the singlet oxygen generation by Tf-IR780 NPs and free IR780. 200 μL Tf-IR780 NPs and 20 μL SOSG (50 μM) were added into a 96-well plate, followed by 808 nm wavelength laser (1 W/cm^2^) irradiation. SOSG fluorescence was excited with a light resource of 504 nm wavelength and an emission of 525 nm using a multifunctional microplate reader (Safire, TECAN). The fluorescence intensity was measured at every 20 seconds for 2 minutes.

### *In vitro* cellular uptake

CT26 and L929 cells were seeded onto a glass-bottom dish (glass diameter 10 mm) at 1.0 × 10^4^ cells/dish and cultured for 24 h at 37 °C in 5% humidified CO_2_. After 24 h, the medium was changed to the fresh medium containing (a) Tf-IR780 NPs (5 μg/mL IR780) for CT26 cells, (b) Tf-IR780 NPs (5 μg/mL IR780) plus free transferrin (10 mg/mL) for CT26 cells, (c) Tf-IR780 NPs (5 μg/mL IR780) for L929 cells, (d) Tf-IR780 NPs (5 μg/mL IR780) plus free transferrin (10 mg/mL) for L929 cells. After 2 h incubation, the cells were washed thrice with PBS and fixed with 4% paraformaldehyde solution for 20 min, then stained with DAPI for 15 min. Finally, the cells were observed by confocal laser scanning microscope (CLSM, Leica TCS SP5, Germany). The excitation wavelength of IR-780 was 633 nm and the emission spectrum recorded was between 700–800 nm.

Cell uptake was further quantitatively analyzed with NIR Imaging. CT26 cells (5 × 10^3^ cells/well) were seeded in 100 μL of culture medium on 96-well plates. Tf-IR780 NPs (5 μg/mL IR780) incubated with CT26 cells for 10 min, 30 min, 60 min, 120 min and 240 min at 37 °C. Near-infrared (NIR) fluorescence images were then detected with an *in vivo* imaging system (IVIS Lumina XR III, USA). The excitation wavelength of IR-780 is 735 nm and emission spectrum of it is 780–900 nm.

### Photothermal and photodynamic toxicity of Tf-IR780 NPs *in vitro*

The cytotoxicity of Tf-IR780 NPs under light irradiation in CT26 cells was determined via CCK-8 assay. Briefly, the cells were seeded in a 96-well plate at a density of 5 × 10^3^ cells/well in 100 μL DMEM containing 10% FBS. After incubated for 24 h, samples with various concentrations of Tf-IR780 NPs were added to each well. 2 h later, the medium was replaced with 200 μL fresh medium and the cells were irradiated with a 1 W/cm^2^ 808-nm laser for 5 min. Control cells were treated identically but not irradiated with the laser. Subsequently, the cells were incubated for 24 h, and CCK-8 assay was used to quantify the cell viability. The cell viability was defined as the percentage of surviving cells versus untreated cells.

To observe the phototherapeutic efficacy of Tf-IR780 NPs directly, the cells irradiated with/without laser were washed with PBS and fixed with 4% paraformaldehyde solution. The cells were stained with calcein-AM for visualization of live cells and with PI for visualization of dead/late apoptotic cells, according to the manufacturer’s suggested protocol (Invitrogen). The cells were observed with Nikon biological inverted microscope (Eclipse, Japan).

### ROS generation by Tf-IR780 NPs in CT26 cells

ROS formation in the CT26 cells was monitored by fluorescence microscopy using H_2_DCFDA as a fluorescent probe. Briefly, 5 × 10^3^ CT26 cells were seeded in a 96-well plate. After 24 h, PBS and Tf-IR780 NPs with or without laser irradiation were incubated with CT26 cells for 2 h at 37 °C. The final concentration of IR780 in Tf-IR780 NPs dispersions was 2.5 μg/mL. After the cells were washed 3 times using PBS, H2DCFDA (40 μM) solution was added into the cells for 0.5 h. Afterward, the cells were rinsed again with PBS, and subsequently illuminated using an 808 nm laser with energy density of 1 W/cm^2^ for 5 min. Fluorescence images of different groups in the irradiated region were immediately captured on a fluorescence microscope using an excitation of 504 nm and an emission of 510–560 nm.

### Animals and tumor model

Male BALB/c mice were purchased from the Model Animal Research Center of Nanjing University and all animals received care in compliance with the guidelines outlined in the Guide for the Care and Use of Laboratory Animals and were used in accordance with the regulations of the Institutional Animal Care and Use Committee (IACUC) of Nanjing University. All animal tests and experimental procedures were approved by the Administration Committee of Experimental Animals in Jiangsu Province and the Ethics Committee of Nanjing University. To establish the tumor model, CT26 cells (5 × 10^6^) were administered by subcutaneous injection into the right flank of the mice. Treatments were initiated with the tumor volumes reached 100–200 mm^3^.

### *In vivo* pharmacokinetics of Tf-IR780 NPs

The tumor-bearing BALB/c mice were used to determine the pharmacokinetics of Tf-IR780 NPs (n = 5). The mice were intravenously (i.v.) injected with 100 μL of Tf-IR780 NPs (12 mg/kg). Blood samples (~100 μL) were drawn from orbital vein at different time points (1, 2, 4, 6, 8, 12, 24, 48 and 72 h). The concentration of IR780 in the blood was determined by the UV–vis absorption using NanoDrop2000 (Thermo Scientific, USA). Blood samples from untreated mice were used as controls to determine the background absorption levels. A series of dilutions of free IR780 were measured to obtain a standard calibration curve. The concentration of the IR780 in the blood samples was determined according to standard curve. PK Solver Version 2.0, was used to calculate pharmacokinetic parameters from the plasma concentration versus time data^30^.

### *In vivo* imaging and biodistribution analysis

When the size of mice tumor reached about 100–200 mm^3^, the Tf-IR780 NPs (0.3 mg IR780/kg body weight) were injected via tail vein (n = 6). Images were taken at 2, 12, 24, 48 and 72 h after injection using the *in vivo* imaging system (IVIS Lumina XR III, USA). Three of the BALB/c mice were sacrificed at 24 h after injection. Then the organs including heart, liver, spleen, lung, kidney, brain and tumor were collected for imaging and semi-quantitative biodistribution analysis by the imaging system. The excitation wavelength of IR-780 is 735 nm and emission spectrum of it is 780–900 nm.

### *In vivo* thermal imaging

When the tumor size reached about 200 mm^3^, 100 μL of Tf-IR780 NPs (20 mg/kg, concentration of IR780) was injected into the tumor-bearing mice by vein. 24 hours later, thermal imaging was taken by a VT02 infrared camera (Fluke, USA) when the tumors were exposed to 808 nm laser of power density at 1 W/cm^2^ at 1 min intervals for a total of 5 min.

### Antitumor activity *in vivo*

When the tumor volumes reached about 100–200 mm^3^, the mice were divided into four groups and treated with PBS, PBS plus NIR laser irradiation, Tf-IR780 NPs (IR780 dose: 20 mg/kg), and Tf-IR780 NPs (20 mg/kg IR780) plus NIR laser irradiation. All samples were given by i.v. injection via tail vein. The day of administration was set as day 0 and 24 hours later, the tumors were exposed to 808 nm laser (1 W/cm^2^) for 5 min. Tumor volumes were calculated using the following formula: tumor volume = length × width^2^ × 0.5. Tumor sizes and body weights were measured every 2 days for the duration of the experiment. Comparative tumor volumes were calculated as V/V0, where V0 is the original tumor volume before the treatment was started.

After more than two weeks of treatments, the animals treated with Tf-IR780 NPs plus NIR laser irradiation were sacrificed and main organs (heart, liver, spleen, lung and kidney) were harvested for Hematoxylin-Eosin (H&E) staining. Tumors harvested from all four groups above were also stained by H&E at 24 h after treatment. H&E slides were observed by a Nikon biological inverted microscope (Eclipse, Japan).

### Statistical Analysis

Unless stated otherwise, all results are shown as mean ± SD. Statistical analyses of data were done using Student’s t test. Here, a single asterisk (*) indicated P < 0.05 and a double asterisk (**) indicated P < 0.01.

## Results and Discussion

**Figure 1:**
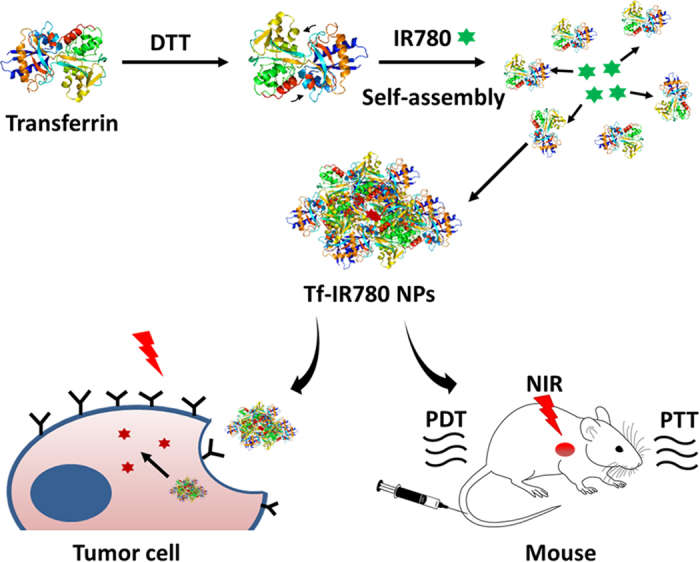
Schematic of Tf-IR780 NPs preparation and the phototherapy mediated by Tf-IR780 NPs. Transferrin exposed its hydrophobic area owing to dithiothreitol (DTT), and interacted with IR780 to form nanoparticles by hydrophobic interaction. Tf-IR780 NPs still have transferrin activity and were endocytosed by cancer cells via transferrin receptor-mediated pathway. Thus, Tf-IR780 NPs accumulated effectively in the tumor of mice and ablated them by combined PDT and PTT.

Tf-IR780 NPs were prepared by a modified “molecular switch” method^31^,^32^,^33^,^34^,^35^,^36^. Transferrin could expose its hydrophobic area following disulfide reduction with DTT, which then enabled IR780 binding by hydrophobic interactions. We hypothesized that the self-assembling process will not affect the binding activity of transferrin to transferrin receptor and that the prepared nanoparticles will exhibit active tumor-targeting. Tf-IR780 NPs could be excited under 808 nm laser irradiation to produce both heat and ROS, killing cancer cells both *in vitro* and *in vivo* (Fig. 1).

### Preparation and characterization of Tf-IR780 NPs

**Figure 2:**
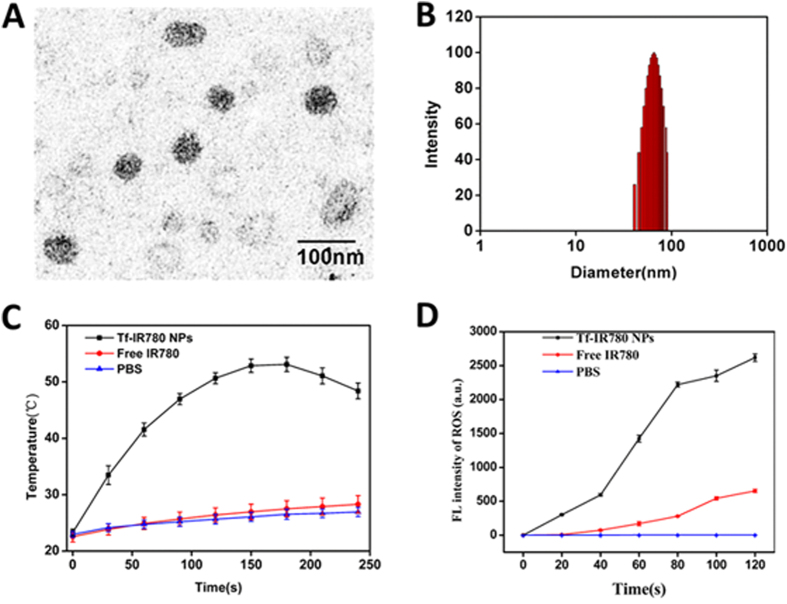
Characterization of Tf-IR780 NPs. (**A**) TEM image of Tf-IR780 NPs; (**B**) The size distribution of Tf-IR780 NPs; (**C**) Temperature curves of Tf-IR780 NPs and free IR780 under exposure to the 808 nm light (1 W/cm^2^) over a period of 5 min; (**D**) ROS generation of Tf-IR780 NPs under exposure to the 808 nm light (1 W/cm^2^) for 2 min, C_IR780_ = 50 μg/mL.

Transmission electron microscopy (TEM) showed that Tf-IR780 NPs had a spherical morphology with sizes less than 100 nm (Fig. 2A). The dynamic light scattering (DLS) measurement obtained similar results. The size distribution of nanoparticles was in 45–90 nm with average hydrodynamic diameter of 65 nm. It was reported that nanoparticles smaller than 100 nm are well-suited for systemic delivery to tumors^37^. The drug loading of Tf-IR780 NPs was 2.3%, as measured by UV/vis absorption spectra.

Following preparation and basic characterization, we then investigated the photothermal profile of the nanoparticles. Free IR780 exhibited different photothermal profile in pure ethanol and DMSO-water mixtures (Fig. S1). While free IR780 ethanol solution exhibited high heat conversion efficiency, IR780 in aqueous solution failed to be adequately activated by the 808 nm laser (1 W/cm^2^), leading to low photothermal heating efficiency. This was because IR780 was fully dissolved in ethanol but aggregated in aqueous solutions. In marked contrast, the temperature of aqueous solution of the Tf-IR780 NPs reached 46.9 °C in 90 s under laser irradiation. The maximum reached temperature was 52.9 °C. Such observed temperatures are high enough to cause significant hyperthermia damage in the therapy area of the body (above 42 °C)^38^. IR780 encapsulated in our nanoparticles can also enhance ROS generation in the same manner as heat effect^15^,^39^. The ROS generation of Tf-IR780 NPs was confirmed by the fluorescence intensity of SOSG. As shown in Fig. 2D, the ROS generation from Tf-IR780 NPs increased during the laser irradiation, indicating continuous generation of singlet oxygen by the Tf-IR780 NPs. The above results demonstrated that Tf-IR780 NPs could be used as both photothermal and photodynamic agent for simultaneous PTT/PDT therapy triggered by a single NIR laser.

**Figure 3:**
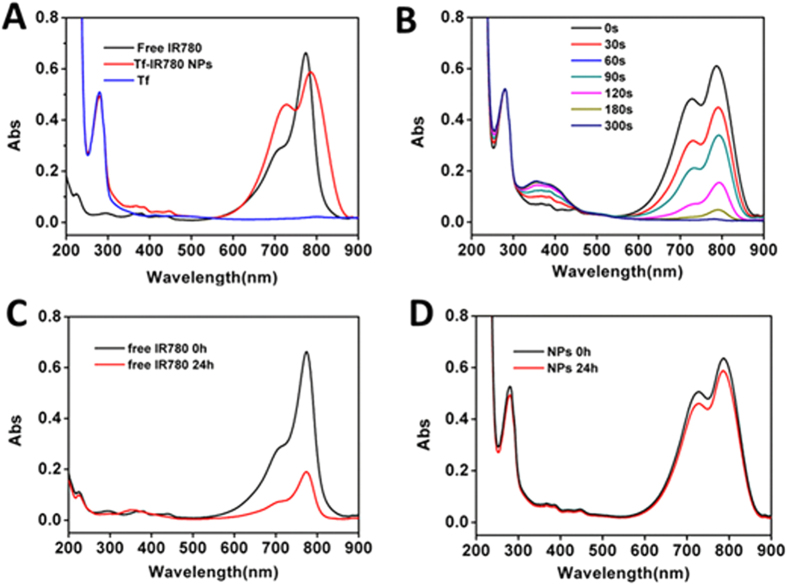
UV/vis absorption spectra of Tf-IR780 NPs and free IR780 under different conditions. (**A**) UV/vis absorption spectra of free IR780, transferrin and Tf-IR780 NPs; (**B**) The absorbance curves of Tf-IR780 NPs after repeated laser irradiation for 5 min; (**C**) Photo-stability of free IR780 under daylight in 24 h; (**D**) Photo-stability of Tf-IR780 NPs under daylight in 24 h.

We next investigated the optical behavior of Tf-IR780 NPs under various conditions. UV/vis absorbance was employed to show the characteristic peak of free IR780, transferrin and Tf-IR780 NPs. After forming Tf-IR780 NPs, the absorption peak of IR780 and transferrin were still present, which proved that hydrophobic IR780 was loaded successfully in transferrin (Fig. 3A). The absorption peak of IR780 had a 7 nm red-shift to 785 nm, probably due to the hydrophobic interactions between transferrin and IR780 in the used polar solvent. The broad absorption (from 600 to 900 nm) of the Tf-IR780 NPs is a benefit for the *in vivo* NIR imaging and photothermal performance^40^. To assess the degradation of Tf-IR780 NPs, the UV-vis spectra were recorded after repeated irradiation. With the increase of irradiation time, peak absorption of IR780 in Tf-IR780 NPs significantly decreased, while the absorption of transferrin was not changed (Fig. 3B). The degradation of IR780 was after forming nanoparticles, IR780 could transfer energy to the oxygen in aqueous solution under NIR laser condition, and generated singlet oxygen which could degrade IR780 into dioxetanes and even into several carbonyl compounds^41^. We speculate that the degradation of IR780 under laser irradiation may be beneficial for reducing the toxicity of IR780 *in vivo* after phototherapy.

To evaluate the photo-stability of Tf-IR780 NPs, we tested the UV-vis spectra of Tf-IR780 NPs at 0 hour or 1 day of storage in light. From the results, the maximum absorbance of free IR780 declined significantly, while the spectra of Tf-IR780 NPs changed only slightly (Fig. 3C,D). Transferrin NPs could partially protect IR780 from photolysis and aggregation in aqueous solution, which made them good candidates for further development in phototherapy applications *in vitro* and *in vivo*.

The *in vitro* release of IR780 from Tf-IR780 NPs is shown in Fig. S3 up to 48 h at the pH values of 5.5, 6.8 and 7.4. From the results, there is no significant difference among the groups at different pH values and only about 12% IR780 could be released from the nanoparticles in 48 h. Due to the sustained release profile of Tf-IR780 NPs, they may intact and display good PTT/PDT effect under laser in different environmental conditions.

### Cell uptake and Phototherapy of Tf-IR780 NPs

**Figure 4:**
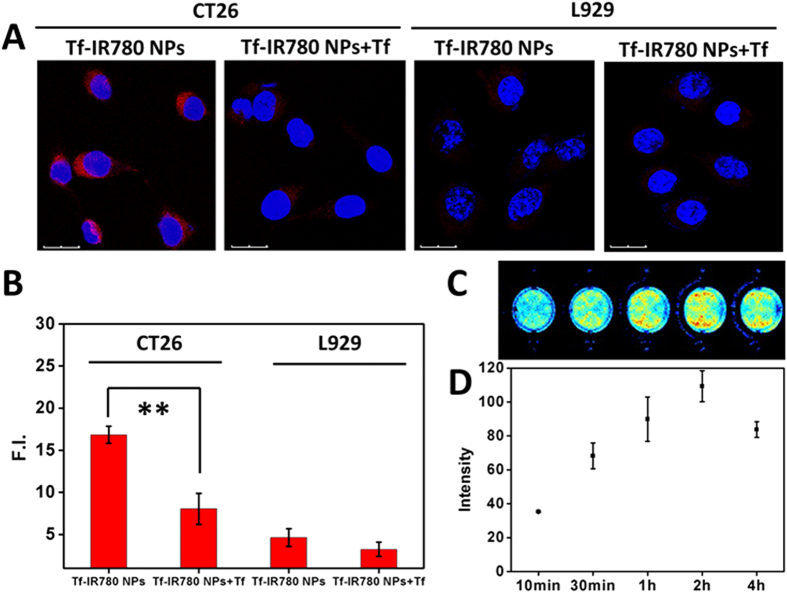
Cell uptake of Tf-IR780 NPs. (**A**) Confocal fluorescence images of CT26 and L929 localization of Tf-IR780 NPs with/without adding transferrin after 2 h incubation. Blue fluorescence indicates positive staining of DAPI, and red fluorescence is the fluorescence of IR780. Scale bar = 20 μm. (**B**) Mean fluorescence intensity of IR780 in each group calculated by Image J; (**C**) The NIR images of Tf-IR780 NPs incubated with CT26 cells for different time; (**D**) Mean fluorescence intensity of IR780 calculated by Image J.

The cell uptake of Tf-IR780 nanoparticles was tested by a confocal fluorescence microscopy using CT26 and L929 cell lines^42^. After 2 h incubation, compared with normal cells (fibroblasts L929), Tf-IR780 NPs treated colon cancer cells (CT26) presented a significantly stronger red color in the cytoplasm, suggesting different uptake behavior between normal and malignant cells (Fig. 4A). To further demonstrate active tumor-targeting ability of the Tf-IR780 NPs, competitive binding experiments using transferrin were performed. As shown in Fig. 4A,B, after treating with free transferrin, red color of IR780 in the cytoplasm of CT26 cells decreased (16.8 vs. 8.0). However, there was no obvious fluorescence intensity difference (4.6 vs. 3.2) in normal cells (L929). Transferrin receptor is overexpressed on the surface of most cancer cells instead of normal cells^43^. The different uptake of Tf-IR780 NPs between CT26 and L929 cell lines may be due to the binding of Tf-IR780 NPs to transferrin receptor and subsequent uptake by tumor cells. The influence of CT26 cells uptake on the fluorescence change of Tf-IR780 NPs was observed by NIR imaging system for different incubation time (Fig. 4C,D). For the first two hours, the uptake of Tf-IR780 NPs increased by time-depended manner, and decreased at the fourth hour. The results indicated that two-hour incubation of Tf-IR780 NPs with CT26 cells was optimal for cell phototherapy.

The results above demonstrated that the encapsulation of IR780 by transferrin to form nanoparticles could improve cell uptake ability, and enhance cancer cell targeting.

**Figure 5:**
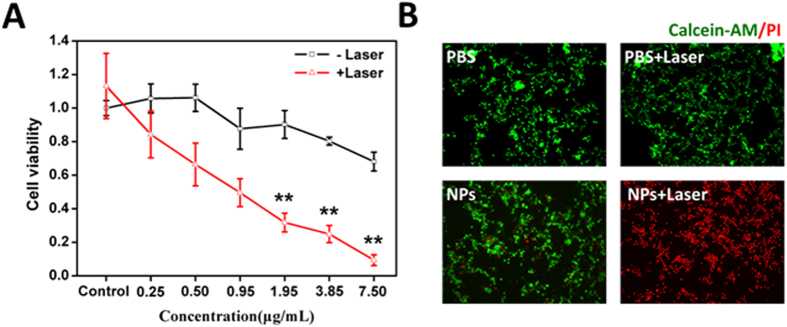
*In vitro* phototherapy of Tf-IR780 NPs. (**A**) Relative viabilities of CT26 cells incubated with different concentrations Tf-IR780 NPs under exposure to the 808 nm laser at the power density of 1 W/cm^2^ (**p < 0.01 ); (**B**) Fluorescence images of CT26 cells with the treatments of PBS, PBS plus laser, Tf-IR780 NPs or Tf-IR780 NPs plus laser. The IR780 concentration was 10 μg/mL. Viable cells were stained green with calcein-AM, and dead cells were stained red with PI.

Then, we investigated the phototherapy effect of Tf-IR780 NPs in CT26 cells. CT26 cells were incubated with different concentrations of Tf-IR780 NPs for 2 h and then irradiated by the 808 nm laser for 5 min. The groups treated with Tf-IR780 NPs at the same concentrations without irradiation were set as controls. Cell viability assay was conducted to investigate the efficiency of the phototherapy (Fig. 5A). When exposed to laser irradiation (1 W/cm^2^), Tf-IR780 NPs induced obvious cytotoxicity, especially at the IR780 concentrations of 1.95, 3.85 and 7.5 μg/mL. In order to visually estimate the *in vitro* therapeutic effect of Tf-IR780 NPs, the CT26 cells were stained by calcein-AM and propidium iodide (PI) to identify live and dead cells, respectively (Fig. 5B). Strong green fluorescence was observed in CT26 cells treated by PBS, PBS plus laser and Tf-IR780 NPs. In contrast, under the NIR laser irradiation most CT26 cells treated by nanoparticles were dead and showed intense red fluorescence signals. This result was consistent with cytotoxicity assay above. The Tf-IR780 NPs in cancer cells could induce ROS and local hyperthermia generation under the laser irradiation. Photodynamic effect of Tf-IR780 NPs was proved by singlet oxygen generation in CT26 cells using H2DCFDA staining (Fig. S2). CT26 cells treated by Tf-IR780 NPs plus NIR irradiation exhibited obvious green fluorescence (H2DCFDA), demonstrating ROS generation in cells. The above results demonstrate that Tf-IR780 NPs could display more toxicity to tumor cells under NIR irradiation. Due to the effects of PTT and PDT under single laser condition, which have been proven in aqueous solution and cell experiments, the Tf-IR780 NPs can greatly kill the cancer cells by both heat and ROS.

### *In Vivo* NIR imaging and Photothermal effect of Tf-IR780 NPs

**Figure 6:**
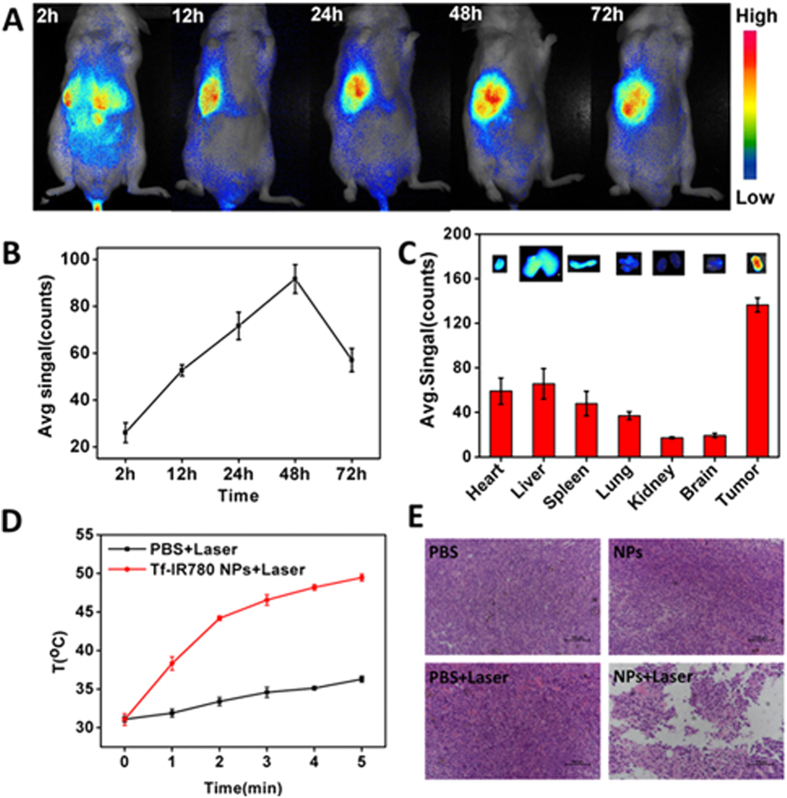
*In vivo* fluorescence imaging and photothermal effects of Tf-IR780 NPs in tumor-bearing mice. (**A**) *In vivo* NIR imaging and (**B**) NIR intensities of the mice bearing CT26 tumor injected with Tf-IR780 NPs (0.3 mg/kg, IR780) at 2, 12, 24, 48 and 72 h post-injection, respectively; (**C**) *Ex vivo* imaging and NIR intensities of Tf-IR780 NPs in heart, liver, spleen, lung, kidney, brain and tumor of the mice bearing CT26 tumor at 24 h post-injection; (**D**) Photothermal effects of Tf-IR780 NPs *in vivo* of CT26 tumor-bearing mice exposed to 808 nm laser for 5 min (1 W/cm^2^). (**E**) H&E stained images of tumor sections collected from different treated groups of mice.

Since IR780 could be used as NIR probe for *in vivo* imaging, the *in vivo* biodistribution profiles of Tf-IR780 NPs were monitored by detecting its fluorescence in CT26 tumor at different time points. As shown in Fig. 6A, the fluorescence signal of IR780 concentrated in the liver as soon as 2 h after injection. At 12 h post-injection, a strong signal was detected in the tumor area with Tf-IR780 NPs and the tumor signal reached a peak at 48 h post-injection (Fig. 6A,B). Quantification of fluorescent signals in the tumor area confirmed tumor accumulation of Tf-IR780 NPs (Fig. 6B). We also observed the distribution of Tf-IR780 NPs in major organs from the *ex vivo* images after 24 h injection. From both the image and the quantification of fluorescent signals, the accumulation of IR780 in tumor was much higher than that in the liver and other examined organs (Fig. 6C). Under the guidance of *in vivo* NIR imaging, we measured the intratumoral temperature *in vivo* at 24 h after intravenous injection of PBS, and Tf-IR780 NPs with exposure to a NIR laser (Fig. 6D). The tumors treated with Tf-IR780 NPs had a maximum temperature of 49.5 °C, which was high enough to induce irreversible tumor tissue damage. In contrast, the temperature of tumors treated with PBS increased only to 36.3 °C, which had no effect on tumor growth or tumor histology. Afterwards, H&E staining of tumor sections was performed to investigate the antitumor effect of Tf-IR780 NPs (Fig. 6E). PBS plus laser group showed no obvious influence on the tumors because of low temperature increase under a NIR laser. These results indicated that the Tf-IR780 NPs could effectively accumulate in tumor tissues and cause significant photothermal effect, which are the prerequisites for successful phototherapy *in vivo*.

### *In vivo* pharmacokinetics and therapeutic efficacy of Tf-IR780 NPs

**Figure 7:**
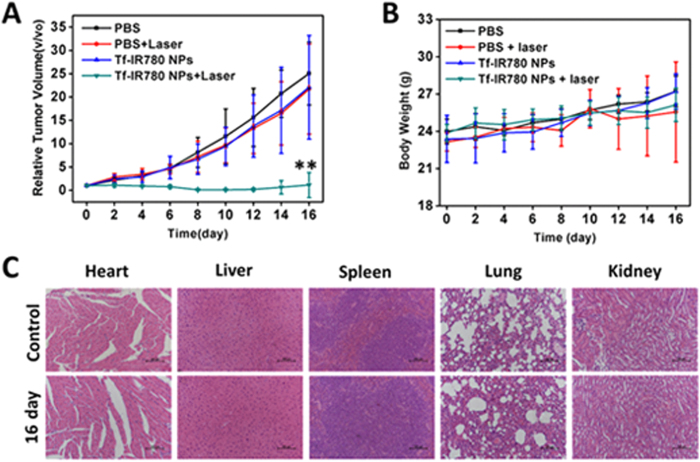
*In vivo* cancer phototherapy in mice models with CT26 cancer cells. (**A**) Tumor growth curves of different groups of CT26 tumor-bearing mice; (**B**) Body weight of mice in different groups after treatment; (**C**) H&E stained images of the heart, liver, spleen, lung and kidneys, from untreated healthy mice and treated mice with Tf-IR780 NPs injection taken 16 days after phototherapy. No noticeable abnormality was observed in major organs. (Scale bar = 100 μm).

We studied the pharmacokinetics of Tf-IR780 NPs in tumor-bearing mice (Fig. S4). The prolonged blood circulation time (t_1/2 _= 20.12 ± 3.76 h) of Tf-IR780 NPs was attributed to the biocompatible transferrin carrier and consistent with its improved stability in aqueous solutions (Table S1). The phototherapeutic efficacy of Tf-IR780 NPs was next evaluated in Balb/c mice bearing subcutaneous CT26 tumors. When the tumors reached 100~200 mm^3^, mice were intravenously injected with PBS or Tf-IR780 NPs. At 24 h post-injection, the mice were irradiated with 808 nm laser (1.0 W/cm^2^) for 5 min. The groups treated with PBS or PBS plus laser irradiation showed rapid tumor growth, suggesting that the laser irradiation alone had no therapeutic benefits (Fig. 7A). The growth of CT26 tumors was significantly inhibited by the Tf-IR780 NPs plus laser irradiation, which indicated that the PTT and PDT effect of Tf-IR780 NPs could effectively suppress tumor growth (Fig. 7A). We also examined the potential toxic side effect of Tf-IR-780 NPs treatment. In the present study, the loss of body weight was analyzed as an indicator for treatment-induced toxicity. No obvious body weight variation was noticed in mice after various treatments (Fig. 7B). We further investigated the potential toxicity of Tf-IR780 NPs combined with laser irradiation to major organs, such as heart, liver, spleen, lung and kidneys. H&E staining of these organs showed no obvious damage 16 days after phototherapy of the Tf-IR780 NPs (Fig. 6C). These data suggested that the phototherapy *in vivo* with Tf-IR780 NPs treatment by single NIR laser did not cause significant adverse effect.

Protein-based drug delivery systems have a promising prospect for clinical use as indicated by the success of albumin-based Abraxane^®^, which has already been approved by the US Food and Drug Administration (FDA) for the treatment of various cancers. Transferrin is another abundant protein in the blood. In the past few decades, transferrin mediated drug delivery has been widely used for targeting delivery of drugs to tumor^43^. Compared with albumin-based nanoparticles, our transferrin nanoparticles showed more effective targeting ability. Jiang *et al*. used human serum albumin as carrier to load IR780, however, HSA-IR780 NPs showed less anti-tumor effect by intravenous plus intratumoral injection because albumin is a nontargeting protein which cannot specifically deliver drugs to tumor^44^. Therefore, there may be good potential for clinical translation of Tf-IR780 NPs, which were prepared by simple method and displayed obvious antitumor effect *in vivo* through PTT and PDT by a single NIR laser irradiation.

## Conclusion

In summary, we have developed a novel theranostic drug delivery system based on hydrophobic IR780-loaded transferrin nanoparticles for cancer imaging and PDT/PTT synergetic phototherapy by a single NIR laser irradiation. Results show that Tf-IR780 NPs are stable in aqueous condition under light condition and exhibit enhanced uptake to cancer cells compared with normal cells. The Tf-IR780 NPs could be simultaneously utilized for NIR imaging and phototherapy both *in vitro* and *in vivo* for transferrin-overexpressed tumors. We propose that our nanoparticles can be a promising strategy for image-guided cancer phototherapy with a great potential for clinical translation.

## Additional Information

**How to cite this article**: Wang, K. *et al*. Self-assembled IR780-loaded transferrin nanoparticles as an imaging, targeting and PDT/PTT agent for cancer therapy. *Sci. Rep.* **6**, 27421; doi: 10.1038/srep27421 (2016).

## Supplementary Material

Supplementary Information
