# Supplementary material for: Self-assembled IR780-loaded transferrin nanoparticles as an imaging, targeting and PDT/PTT agent for cancer therapy
Source: Sci Rep. 2016 Jun 6;6:27421. doi: 10.1038/srep27421 (PMC4899881; doi:10.1038/srep27421)
Supplement: Supplementary Information [file srep27421-s1.doc]

**Supplementary materials**

**Self-assembled IR780-loaded transferrin nanoparticles as an imaging, targeting and PDT/PTT agent for cancer therapy**

Kaikai Wang, Yifan Zhang, Juan Wang, Ahu Yuan, Minjie Sun, Jinhui Wu, Yiqiao Hu.

Figure S1. Photothermal performance of free IR780 dissolved in different solvents (pure ethanol, DMSO-water and ethanol-water) under 808nm laser irradiation for 5 min.

**
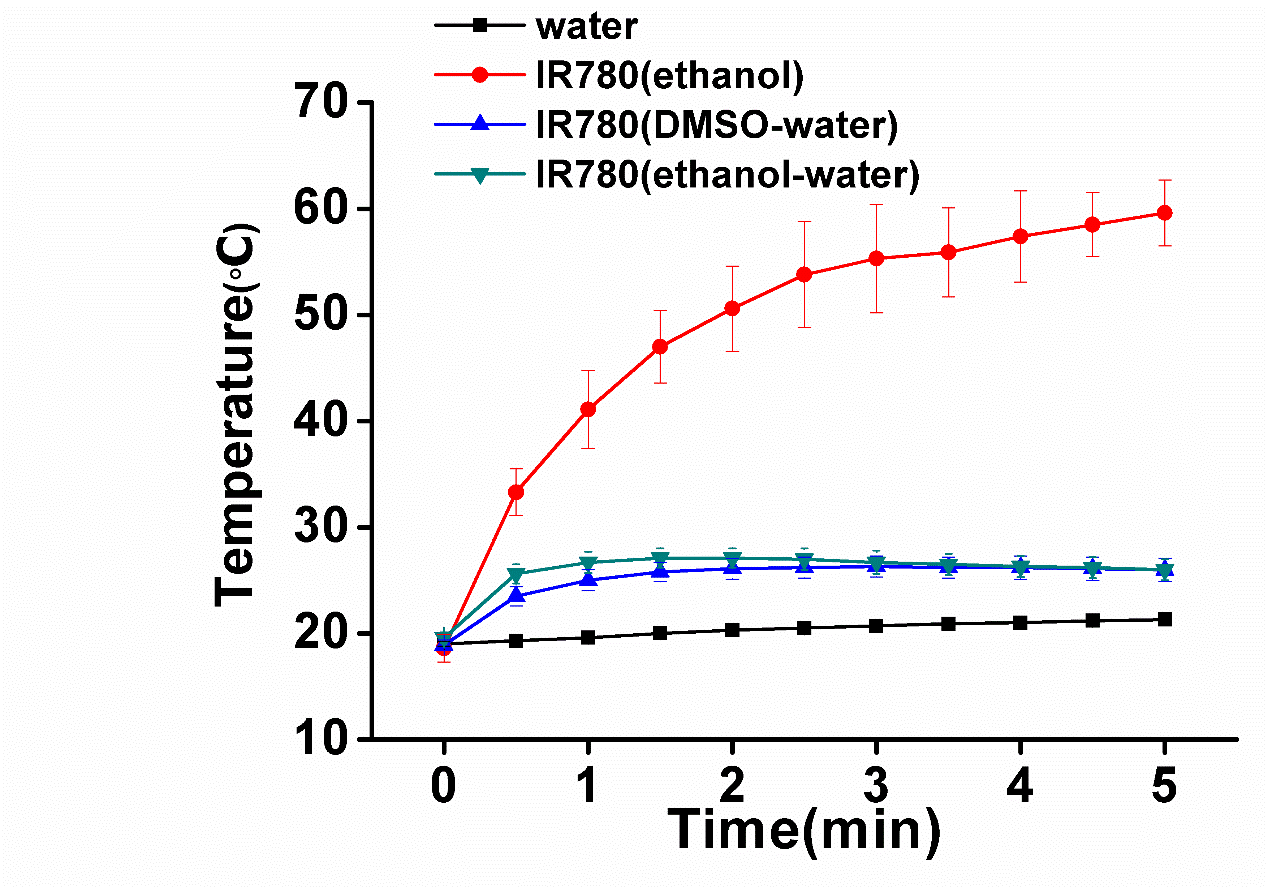
**

Figure S2. Fluorescence images of CT26 cells treated with PBS, PBS plus laser, Tf-IR780 NPs or Tf-IR780 NPs plus laser using H_2_DCFDA staining for singlet oxygen detection under photoirradiation (1 W/cm^2^, 808 nm) for 5min. (Scale bar = 20 μm.)


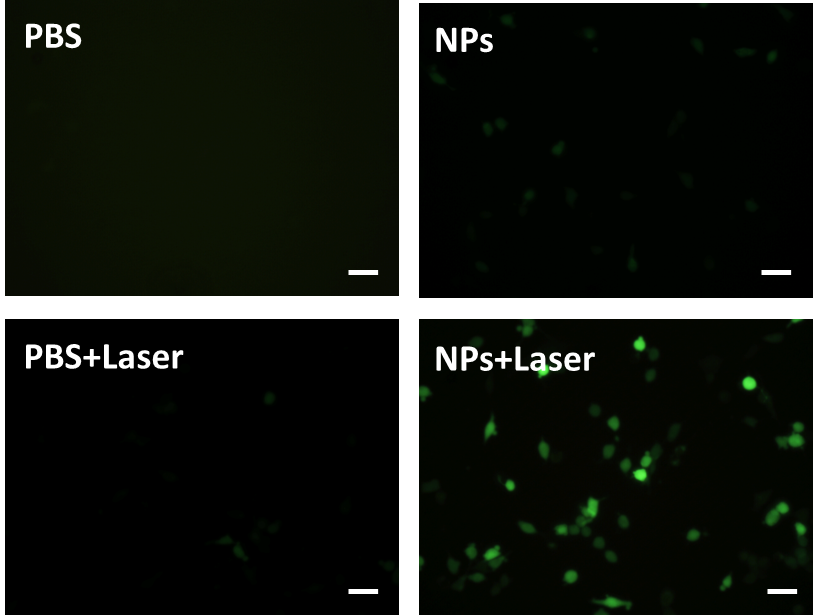


Figure S3. In vitro release profiles of IR780 from the transferrin nanoparticles at different pH values at 37^o^C.


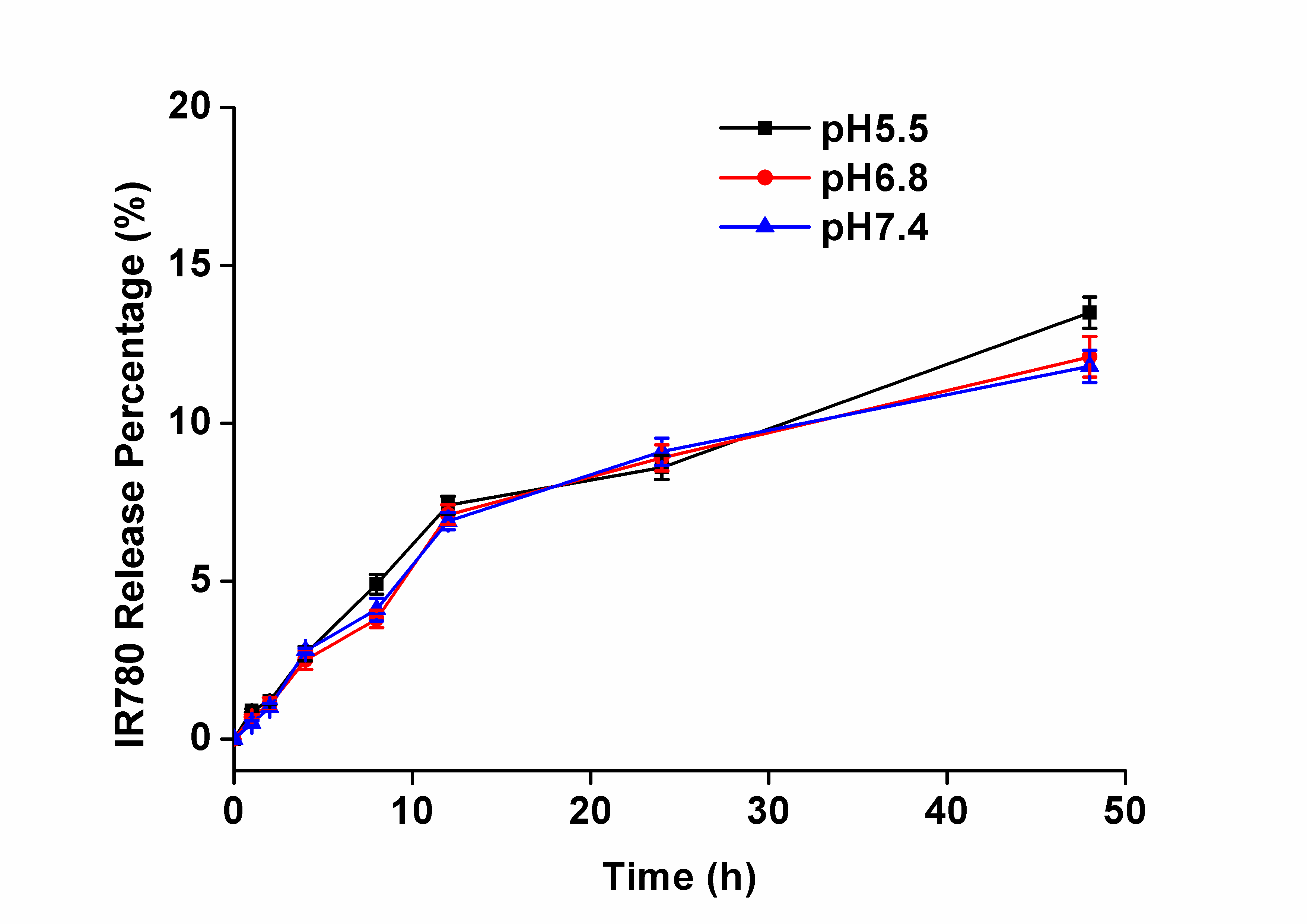


Figure S4. Pharmacokinetics of Tf-IR780 NPs in mice after intravenous injection determined based on IR780 absorption. A) IR780 standard curve used to calculate plasma concentration levels of treated mice. B) Blood circulation curves of Tf-IR780 NPs at interval times.


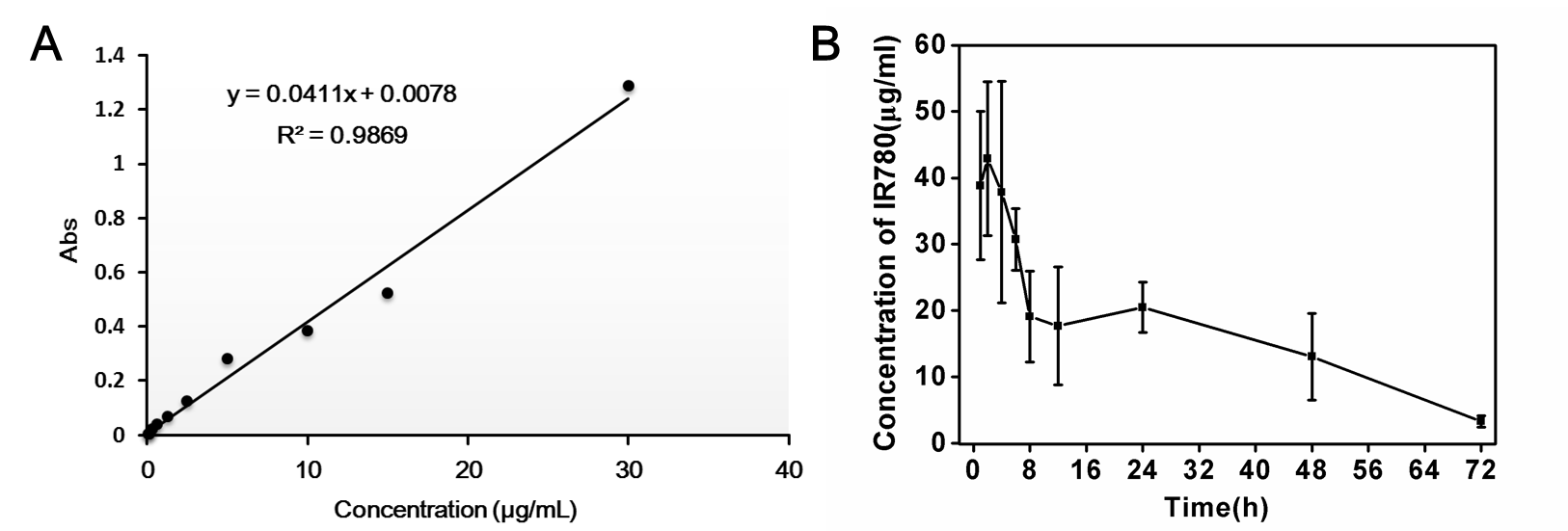


**Table S1** Pharmacokinetic parameters of Tf-IR780 NPs in CT-26 cells bearing mice.

| **Pharmacokinetic Parameters** | **Unit** | **Tf-IR780 NPs** |
| --- | --- | --- |
| **t_1/2_** | **h** | **20.1156±3.7589** |
| **AUC** | **μg/mL*h** | **1022.0752±180.7770** |
| **CL** | **(μg)/(μg/mL)/h** | **0.2199±0.0408** |
| **MRT** | **h** | **29.0207±5.4230** |

**t_1/2_: Half-life; AUC: Area under the curve; MRT: Mean residence time; CL: clearance.**
